# Migraine, vascular risk, and cardiovascular events in women: prospective cohort study

**DOI:** 10.1136/bmj.a636

**Published:** 2008-08-07

**Authors:** Tobias Kurth, Markus Schürks, Giancarlo Logroscino, J Michael Gaziano, Julie E Buring

**Affiliations:** 1 Division of Preventive Medicine, Department of Medicine, Brigham and Women’s Hospital, Harvard Medical School, Boston, MA, USA; 2 Division of Aging, Department of Medicine, Brigham and Women’s Hospital, Harvard Medical School, Boston, MA; 3 Department of Epidemiology, Harvard School of Public Health, Boston, MA; 4 Department of Neurology and Psychiatry, School of Medicine, University of Bari, Italy; 5 Massachusetts Veterans Epidemiology Research and Information Center, Boston VA Healthcare System, Boston, MA; 6 Department of Ambulatory Care and Prevention, Harvard Medical School, Boston, MA

## Abstract

**Objectives** To evaluate whether the association between migraine with aura and increased risk of cardiovascular disease is modified by vascular risk groups as measured by the Framingham risk score for coronary heart disease.

**Design** Prospective cohort study.

**Setting** Women’s health study, United States.

**Participants** 27 519 women who were free from cardiovascular disease at baseline with available information on the Framingham risk score and migraine status.

**Main outcome measures** Time to major cardiovascular disease event (non-fatal myocardial infarction, non-fatal ischaemic stroke, death from ischaemic cardiovascular disease), myocardial infarction, and ischaemic stroke.

**Results** At baseline, 3577 (13.0%) women reported active migraine, of whom 1418 (39.6%) reported migraine with aura. During 11.9 years of follow-up, there were 697 cardiovascular disease events. We stratified participants based on 10 year risk of coronary heart disease estimated from the Framingham risk score (≤1%, 2-4%, 5-9%, and ≥10%). Compared with women without migraine, the age adjusted hazard ratios in women with active migraine with aura were 1.93 (95% confidence interval 1.45 to 2.56) for major cardiovascular disease, 1.80 (1.16 to 2.79) for ischaemic stroke, and 1.94 (1.27 to 2.95) for myocardial infarction. When stratified by Framingham risk score, the association between migraine with aura and major cardiovascular disease was strongest in the lowest risk score group. There was a diametric association pattern for ischaemic stroke and myocardial infarction. Compared with women without migraine, the age adjusted hazard ratios in women who reported migraine with aura in the lowest Framingham risk score group were 3.88 (1.87 to 8.08) for ischaemic stroke and 1.29 (0.40 to 4.21) for myocardial infarction. Hazard ratios in women with migraine with aura in the highest Framingham risk score group were 1.00 (0.24 to 4.14) for ischaemic stroke and 3.34 (1.50 to 7.46) for myocardial infarction. Women with migraine without aura were not at increased risk of ischaemic stroke or myocardial infarction in any of the Framingham risk score groups.

**Conclusion** The association between migraine with aura and cardiovascular disease varies by vascular risk status. Information on history of migraine and vascular risk status might help to identify women at increased risk for specific future cardiovascular disease events.

**Trial registration** Clinical trials NCT00000479.

## Introduction

Migraine is a common primary headache disorder characterised by severe, pulsating, mostly unilateral headaches accompanied by vomiting, nausea, and autonomic dysfunctions. In the United States alone, about 18% of the female population and 6% of the male population have migraine headaches.^1^ Some people experience transient neurological symptoms before or during the headache that are most often visual, such as seeing zigzag lines or flash spots, but might also include sensory or motor symptoms. These symptoms are known as migraine aura. The pathophysiology of migraine involves the neuronal system as well as the cranial vascular system.^2^ Peripheral arteries can also be affected in patients with migraine.^3^ ^4^

Migraine with aura is associated with an increased risk of ischaemic stroke,^5^ ^6^ ^7^ ^8^ angina,^9^ ^10^ and other ischaemic vascular events, including myocardial infarction.^11^ ^12^ It remains unclear which mechanisms link migraine with vascular events and whether the biological mechanisms leading to ischaemic stroke differ from the mechanisms leading to myocardial infarction.

Migraine with aura has also been associated with an unfavourable cardiovascular risk profile and increased 10 year predicted risk of coronary heart disease based on the Framingham risk score.^13^ This has led to speculation about whether the increased risk of ischaemic vascular events among patients with migraine with aura is partly caused by this increased prevalence of cardiovascular risk factors. Several observations question this hypothesis. Firstly, the association between migraine and ischaemic vascular events is independent of many cardiovascular risk factors.^11^ ^12^ Secondly, the increased risk of ischaemic stroke is more apparent among individuals without cardiovascular risk factors,^5^ ^7^ ^8^ with the exception of smoking^14^ ^15^ or use of oral contraceptives,^6^ ^14^ ^15^ or both.^8^ A potential association between migraine and cardiovascular disease that depends on the underlying vascular risk status is important for clinicians as it might help to identify individuals at increased risk of future cardiovascular disease events. We evaluated whether the association between migraine with aura and overall and specific cardiovascular disease events differed according to vascular risk status, as measured by the Framingham risk score.^16^

## Methods

### Study population

We carried out a prospective cohort study among participants in the women’s health study, a completed trial designed to test the benefits and risks of low dose aspirin and vitamin E in the primary prevention of cardiovascular disease and cancer among apparently healthy women. The design and results have been described in detail previously.^17^ ^18^ Briefly, 39 876 US female health professionals aged ≥45 at study entry (1992-5) and without a history of cardiovascular disease, cancer, or other major illnesses, were randomly assigned to aspirin (100 mg on alternate days), vitamin E (600 IU on alternate days), both active agents, or both placebos. Baseline information was self reported and was collected by a mailed questionnaire that asked about many cerebrovascular risk factors and lifestyle variables. Twice in the first year and yearly thereafter, participants were sent follow-up questionnaires asking about study outcomes, personal characteristics, medical history, and health habits. For this analysis, we included follow-up information from study entry to 31 March 2007. As of this date, follow-up was 97% complete.

Before randomisation, blood samples were collected in tubes containing EDTA from 28 345 participating women and stored in vapour phase liquid nitrogen (−170°C). Samples were analysed for lipids and inflammatory biomarkers. Total cholesterol, high density lipoprotein cholesterol, and directly obtained low density lipoprotein cholesterol were assayed with reagents from Roche Diagnostics (Basel, Switzerland) and Genzyme (Cambridge, MA). Cholesterol analyses could be performed on 27 939 of the blood samples.

### Assessment of migraine

On the baseline questionnaire participants were asked: “Have you ever had migraine headaches?” and “In the past year, have you had migraine headaches?” From this information, we categorised women into “no history of migraine” and “any history of migraine.” We also distinguished between “active migraine,” which included women with self reported migraine in the year before they completed the baseline questionnaire, and “prior migraine,” which included women who reported ever having had a migraine but none in the year before they completed the questionnaire. Those participants who reported active migraine were asked about migraine specific features. In the women’s health study,^11^ ^19^ we have previously shown good agreement with 1988 International Headache Society (IHS) criteria for migraine.^20^ Specifically, we showed that among those who provided a blood sample and reported active migraine, over 83% fulfilled all but one IHS criterion (code 1.7, migrainous disorder) and over 46% fulfilled all IHS criteria for migraine (code 1.1 migraine without aura).^11^

Participants who reported active migraine were further asked whether they had an “aura or any indication a migraine is coming.” Responses were used to classify women who reported active migraine into active migraine with aura and active migraine without aura.

### Framingham risk score

We used the Framingham risk score, which predicts the 10 year risk of coronary heart disease, as defined by the National Cholesterol Education Program,^16^ to classify participating women into vascular risk classes. This score assigns points for age, total cholesterol, high density lipoprotein cholesterol, smoking, and systolic blood pressure stratified by treatment for hypertension. The points for each individual component are summed and categorised into groups of 10 year risk of coronary heart disease of ≤1% (≤12 points), 2-4% (13-16 points), 5-9% (17-19 points), and ≥10% (≥20 points), as in a previous report of the women’s health study.^21^

### Outcome ascertainment

All participants were followed up for the occurrence of a major ischaemic vascular event, a combined end point defined as the first of any of non-fatal ischaemic stroke, non-fatal myocardial infarction, or ischaemic cardiovascular death. In addition, we evaluated any first myocardial infarction, ischaemic stroke, coronary revascularisation procedure, and angina. There were too few cases of cardiovascular death to perform meaningful stratified analyses. Medical records were obtained for all reported cardiovascular end points except angina and reviewed by an end points committee of physicians. Stroke was confirmed if the participant had a new focal neurological deficit of sudden onset that persisted for more than 24 hours and was then classified as ischaemic, haemorrhagic, or undefined, with excellent agreement between observers.^22^ For this report, we included only cases of ischaemic stroke and censored participants with other subtypes of stroke at the time of event. The occurrence of myocardial infarction was confirmed if symptoms met World Health Organization criteria and if the event was associated with abnormal levels of cardiac enzymes or abnormal results on electrocardiography. Cardiovascular deaths were confirmed by autopsy reports, death certificates, medical records, and information obtained from next of kin or family members.

### Statistical analyses

Of the 27 939 women with completed blood assays, we excluded 79 with missing information on migraine status, 321 with missing information on Framingham risk score, and 20 who reported angina before they received the baseline questionnaire, leaving 27 519 women free from cardiovascular disease or angina at study entry for this analysis. We compared the baseline characteristics of participants, with respect to their migraine status, using analysis of covariance for continuous measurements adjusted for age. We used direct standardisation to adjust categorical variables for age in 5 year increments.

We used multinominal logistic regression models to calculate prevalence odds ratios and 95% confidence intervals of the association between migraine and migraine aura status with Framingham risk score categories, adjusting for age. We used age adjusted Cox proportional hazards models to evaluate the association between migraine and migraine aura status with risks of incident cardiovascular disease or angina. We ran stratified analyses on categories of predicted 10 year risk of coronary heart disease based on Framingham risk score (≤1%, 2-4%, 5-9%, and ≥10%). In addition, we ran stratified analyses of the association between migraine and the various outcomes for individual components of the Framingham risk score (age, systolic blood pressure, smoking, total cholesterol, and high density lipoprotein cholesterol). All analyses were performed with SAS version 9.1 (SAS, Cary, NC); P values were two tailed and P<0.05 was considered significant.

## Results

Of the 27 519 participants, 5074 (18.4%) women reported any history of migraine, and 3577 (13.0%) reported active migraine (migraine in the year before the baseline questionnaire), of whom 1418 (39.6%) reported migraine aura.

Table 1 summarises the association between baseline characteristics according to migraine status. Compared with women without a history of migraine, women with active migraine with aura were younger, less likely to consume alcohol, less likely to exercise regularly, and more likely to currently use hormone therapy after the menopause. Women with prior migraine tended to have raised total cholesterol concentration and were more likely to currently smoke cigarettes, have higher systolic blood pressure, and to have a history of hypertension compared with the other migraine groups. Mean concentration of high density lipoprotein cholesterol was higher among women without a history of migraine. Family history of myocardial infarction was equally distributed among the migraine groups.

**Table 1 tbl1:** Age adjusted baseline characteristics in 27 519 women according to migraine status in women’s health study. Figures are percentages* of women unless stated otherwise

Characteristics	No migraine history	Active migraine with aura	Active migraine without aura	Prior migraine†	P value‡
No of participants	22 445	1418	2159	1497	
Mean (SE) age (years)	54.9 (0.05)	53.2 (0.19)	52.6 (0.15)	55.5 (0.18)	<0.001
Mean (SE) Framingham risk score	12.2 (0.02)	12.3 (0.09)	12.3 (0.07)	12.7 (0.08)	<0.001
Framingham estimate of 10 year risk of coronary heart disease:					
≤1	54.4	53.6	54.3	48.9	<0.001
2-4	30.4	30.3	30.8	32.3	
5-9	11.4	11.8	11.5	13.2	
≥10	3.9	4.3	3.5	5.6	
Mean (SE) total cholesterol (mmol/l)	5.46 (0.002)	5.52 (0.03)	5.51 (0.02)	5.56 (0.03)	0.001
Mean (SE) HDL cholesterol (mmol/l)	1.39 (0.003)	1.37 (0.01)	1.38 (0.008)	1.36 (0.01)	0.001
Mean (SE) systolic blood pressure (mm Hg)	123.6 (0.09)	123.5 (0.35)	124.4 (0.29)	124.6 (0.34)	0.001
Smoking:					
Never	51.2	52.4	56.2	50.5	0.03
Past	37.0	37.3	34.5	35.5	
Current	11.8	10.3	9.3	14.1	
History of hypertension§	24.7	25.6	26.1	30.5	<0.001
History of diabetes	2.5	1.8	1.6	2.6	0.23
Mean (SE) BMI	25.9 (0.03)	25.8 (0.13)	26.2 (0.11)	26.1 (0.13)	0.02
Alcohol consumption:					
Rarely/never	43.5	48.2	47.4	45.1	<0.001
1-3 drinks/month	13.1	12.9	15.4	14.3	
1-6 drinks/week	32.7	30.5	30.0	30.4	
≥1 drink/day	10.8	8.4	7.2	10.3	
Physical activity:					
Rarely/never	36.9	38.0	39.4	39.2	<0.001
<1/week	19.2	20.4	21.7	20.2	
1-3/week	32.3	30.9	29.3	29.4	
≥4/week	11.6	10.7	9.7	11.2	
Hormone therapy after menopause:					
Never	49.4	38.7	44.7	46.0	<0.001
Past	8.7	10.2	8.5	10.2	
Current	41.9	51.2	46.7	43.8	
Family history of myocardial infarction before age 60	12.6	14.4	13.0	14.0	0.09

SE=standard error; HDL=high density lipoprotein; BMI=body mass index.

*Proportions might not add up to 100% because of rounding or missing values.

†Women who indicated history of migraine but no active migraine in year before completing baseline questionnaire.

‡From analysis of covariance for continuous variable or Mantel-Haenszel test for categorical variables.

§Defined as previous diagnosis of hypertension, systolic blood pressure ≥140 mm Hg, diastolic blood pressure ≥90 mm Hg, or receipt of blood pressure lowering treatment at baseline.

With regard to categories of the Framingham risk score, women who reported any history of migraine were more likely to have a higher predicted 10 year risk of coronary heart disease. Compared with women without a history of migraine and low estimated risk for coronary heart disease (≤1%), the age adjusted odds ratio of a predicted 10 year risk of coronary heart disease of ≥10% was 1.26 (95% confidence interval 1.06 to 1.49) for women who reported any history of migraine. After further stratification, this increased risk was apparent only in the group of migraineurs who indicated prior migraine (history of migraine but not active migraine) (1.83, 1.43 to 2.35) (table 2).

**Table 2 tbl2:** Age adjusted association (odds ratio)* between migraine and Framingham predicted 10 year risk of coronary heart disease in 27 519 women in women’s health study

	Framingham estimate of 10 year risk of coronary heart disease											
	No of women in category ≤1%	2-4%				5-9%				≥10%		
		No of women	OR (95% CI)	P value		No of women	OR (95% CI)	P value		No of women	OR (95% CI)	P value
No history of migraine (n=22 445)	12 000	6912	1.00 (reference)	—		2632	1.00 (reference)	—		901	1.00 (reference)	—
History of migraine:												
Any (n=5074)	2862	1487	1.09 (1.01 to 1.17)	0.03		535	1.15 (1.03 to 1.29)	0.02		190	1.26 (1.06 to 1.49)	0.009
Active migraine with aura (n=1418)	840	393	1.02 (0.89 to 1.17)	0.76		141	1.14 (0.94 to 1.40)	0.19		44	1.14 (0.82 to 1.59)	0.42
Active migraine without aura (n=2159)	1332	595	1.02 (0.92 to 1.14)	0.72		178	1.00 (0.84 to 1.20)	0.97		54	1.02 (0.76 to 1.36)	0.92
Prior migraine† (n=1497)	690	499	1.27 (1.12 to 1.45)	<0.01		216	1.46 (1.22 to 1.75)	<0.01		92	1.83 (1.43 to 2.35)	<0.01

*Calculated from multinominal logistic regression model with women without history of migraine and with Framingham estimated 10 year risk of coronary heart disease ≤1% as reference group.

†Women with history of migraine but no active migraine in year before completing baseline questionnaire.

During a mean of 11.9 years of follow-up (326 547 person years), 697 participants had a first major ischaemic cardiovascular disease event, which, taking into account the potential for multiple events in a single individual, included 306 first ischaemic strokes, 301 first myocardial infarctions, and 148 deaths from cardiovascular disease. In addition, 647 underwent coronary revascularisation, and 412 reported angina during follow-up. The risk of major cardiovascular disease increased with increasing Framingham risk score. Compared with women with a predicted 10 year risk of coronary heart disease of ≤1%, the age adjusted hazard ratios for major cardiovascular disease were 3.11 (2.46 to 3.93), 6.04 (4.67 to 7.81), and 10.40 (7.76 to 13.94) for women with predicted risks of 2-4%, 5-9%, and ≥10%, respectively.

Active migraine with aura was associated with about a twofold increased risk of major cardiovascular disease, ischaemic stroke, and myocardial infarction (table 3). After stratification by Framingham risk score groups, the pattern of association between active migraine with aura and major cardiovascular disease was U shaped, with the highest estimate in the lowest risk score group. We observed a strikingly different pattern of association for the outcomes of ischaemic stroke and myocardial infarction (table 3, figure) [Fig fig1]. Compared with women without a history of migraine, the age adjusted hazard ratios for ischaemic stroke were 3.88 (1.87 to 8.08) in the lowest risk score group and 1.00 (0.24 to 4.14) for the highest. For myocardial infarction, these estimates were 1.29 (0.40 to 4.21) and 3.34 (1.50 to 7.46), respectively.

**Figure fig1:**
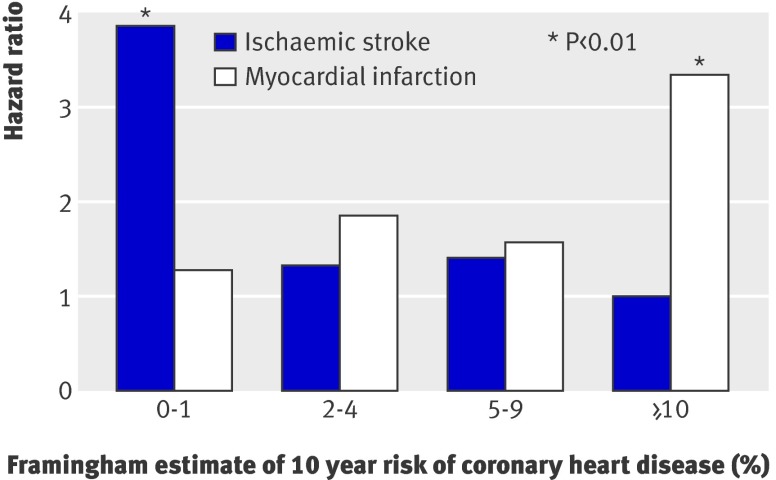
Age adjusted association between migraine with aura and ischaemic stroke and myocardial infarction according to 10 year risk of coronary heart disease estimated by Framingham risk score. Reference group was women without history of migraine

**Table 3 tbl3:** Age adjusted association (hazard ratio) between migraine and cardiovascular disease, stratified by Framingham estimate of 10 year risk of coronary heart disease* in 27 519 women in women’s health study

	No history of migraine (n=22 445)†	Active migraine with aura (n=1418)				Active migraine without aura (n=2159)				Prior migraine‡ (n=1497)		
		No of women	HR (95% CI)	P value		No of women	HR (95% CI)	P value		No of women	HR (95% CI)	P value
**Major cardiovascular event§ (n=697)**												
Overall	557	53	1.93 (1.45 to 2.56)	<0.001		39	1.00 (0.72 to 1.38)	0.98		48	1.23 (0.92 to 1.66)	0.16
Framingham risk group:												
≤1%	80	14	2.69 (1.52 to 4.75)	<0.001		9	1.09 (0.55 to 2.18)	0.80		8	1.72 (0.83 to 3.57)	0.14
2-4%	204	16	1.60 (0.96 to 2.67)	0.07		18	1.22 (0.75 to 1.99)	0.42		11	0.74 (0.40 to 1.36)	0.33
5-9%	169	14	1.80 (1.04 to 3.11)	0.04		9	0.87 (0.45 to 1.71)	0.69		16	1.19 (0.71 to 1.98)	0.51
≥10%	104	9	1.93 (0.97 to 3.82)	0.06		3	0.52 (0.17 to 1.66)	0.27		13	1.32 (0.74 to 2.35)	0.35
**Ischaemic stroke (n=306)**												
Overall	251	22	1.80 (1.16 to 2.79)	0.008		17	0.99 (0.60 to 1.62)	0.97		16	0.91 (0.55 to 1.50)	0.70
Framingham risk group:												
≤1%	36	9	3.88 (1.87 to 8.08)	<0.001		4	1.09 (0.39 to 3.07)	0.87		3	1.44 (0.44 to 4.66)	0.55
2-4%	94	6	1.34 (0.59 to 3.07)	0.48		9	1.39 (0.70 to 2.77)	0.35		3	0.44 (0.14 to 1.38)	0.16
5-9%	77	5	1.41 (0.57 to 3.51)	0.46		2	0.43 (0.11 to 1.75)	0.24		8	1.30 (0.63 to 2.70)	0.48
≥10%	44	2	1.00 (0.24 to 4.14)	0.99		2	0.96 (0.23 to 4.00)	0.95		2	0.49 (0.12 to 2.02)	0.32
**Myocardial infarction (n=301)**												
Overall	240	24	1.94 (1.27 to 2.95)	0.002		17	0.95 (0.58 to 1.56)	0.85		20	1.20 (0.76 to 1.89)	0.44
Framingham risk group:												
≤1%	35	3	1.29 (0.40 to 4.21)	0.67		4	1.10 (0.39 to 3.09)	0.86		3	1.48 (0.45 to 4.80)	0.52
2-4%	83	8	1.87 (0.90 to 3.88)	0.09		9	1.41 (0.71 to 2.83)	0.33		5	0.83 (0.34 to 2.04)	0.68
5-9%	79	6	1.58 (0.69 to 3.65)	0.28		3	0.60 (0.19 to 1.92)	0.39		6	0.94 (0.41 to 2.16)	0.89
≥10%	43	7	3.34 (1.50 to 7.46)	0.003		1	0.35 (0.05 to 2.54)	0.30		6	1.36 (0.58 to 3.20)	0.48

*Women in each risk group: 14 862 in ≤1%; 8399 in 2-4%; 3167 in 5-9%; 1091 in ≥10%.

†Hazard ratio=1.

‡History of migraine but no active migraine in year before completion of baseline questionnaire.

§Defined as first of any of: non-fatal ischaemic stroke, non-fatal myocardial infarction, or death from ischaemic cardiovascular cause.

With regard to the association between active migraine with aura and coronary revascularisation, the hazard ratios were 1.58 (0.80 to 3.14), 1.70 (1.03 to 2.79), 0.93 (0.46 to 1.90), and 2.21 (1.11 to 4.40) for risk score groups ≤1%, 2-4%, 5-9%, and ≥10%, respectively. For angina, these estimates were 2.12 (1.01 to 4.45), 1.40 (0.68 to 2.88), 1.09 (0.48 to 2.50), and 2.06 (0.82 to 5.17).

When we evaluated the individual components of the Framingham risk score, the association between active migraine with aura and ischaemic stroke was particularly apparent for the younger age groups (45-54 and 55-64) and was not apparent among women aged ≥65 (table 4). The risk was further increased for women aged 45-49 (5.35, 2.08 to 13.79; P<0.001). With regard to systolic blood pressure, we found a greater risk of ischaemic stroke for women with low blood pressure. This trend was further magnified among women with systolic blood pressure <110 mm Hg (5.18, 1.70 to 15.80; P<0.004). Furthermore, the associated risk of ischaemic stroke for women with active migraine with aura increased with decreasing total cholesterol concentrations. When we evaluated lower cholesterol concentrations, women with migraine with aura and cholesterol <4.40 mmol/l had further increased risk (4.01, 1.15 to 13.96, P=0.03). This pattern was not driven by high density lipoprotein cholesterol (table 4).[Table tbl4] There was a U shaped pattern with regard to smoking, showing increased risk for current smokers and those who had never smoked, which reached significance for those who had never smoked. 

**Table 4 tbl4:** Age adjusted association (hazard ratio) between migraine and ischaemic stroke, stratified by components of Framingham risk score in 27 519 women in women’s health study

	No history of migraine (n=22 445)*		Active migraine with aura (n=1418)				Active migraine without aura (n=2159)				Previous migraine† (n=1497)		
			No of women	HR (95% CI)	P value		No of women	HR (95% CI)	P value		No of women	HR (95% CI)	P value
Age (years):													
45-54 (n=16 444)	64		12	2.59 (1.40 to 4.80)	0.003		8	1.07 (0.51 to 2.24)	0.85		2	0.51 (0.12 to 2.07)	0.34
55-64 (n=8160)	91		9	2.13 (1.07 to 4.23)	0.03		5	0.79 (0.32 to 1.95)	0.61		8	1.19 (0.58 to 2.46)	0.63
≥65 (n=2915)	96		1	0.32 (0.04 to 2.29)	0.26		4	1.28 (0.47 to 3.49)	0.63		6	0.85 (0.37 to 1.93)	0.69
Systolic blood pressure (mm Hg):													
<120 (n=12 389)	55		10	3.42 (1.74 to 6.75)	<0.001		3	0.69 (0.22 to 2.22)	0.54		4	1.21 (0.44 to 3.34)	0.71
120-139 (n=11 678)	102		6	1.18 (0.52 to 2.69)	0.69		10	1.47 (0.76 to 2.84)	0.25		7	0.93 (0.43 to 2.00)	0.85
≥140 (n=3452)	94		6	1.49 (0.65 to 3.42)	0.34		4	0.65 (0.24 to 1.77)	0.40		5	0.67 (0.27 to 1.65)	0.39
Smoking:													
Never (n=14 219)	104		11	2.31 (1.23 to 4.32)	0.009		8	1.12 (0.54 to 2.32)	0.76		4	0.54 (0.20 to 1.45)	0.22
Past (n=10 100)	98		6	1.23 (0.54 to 2.82)	0.62		5	0.82 (0.33 to 2.03)	0.67		6	0.89 (0.39 to 2.04)	0.79
Current (n=3200)	49		5	2.10 (0.84 to 5.30)	0.11		4	1.25 (0.45 to 3.47)	0.67		6	1.63 (0.70 to 3.81)	0.26
Total cholesterol (mmol/l):													
<5.17 (n=11 171)	58		10	3.29 (1.68 to 6.45)	<0.001		9	2.18 (1.07 to 4.44)	0.03		7	1.85 (0.84 to 4.05)	0.12
5.17-6.20 (n=10 292)	106		5	1.08 (0.44 to 2.65)	0.87		4	0.58 (0.21 to 1.58)	0.29		4	0.58 (0.21 to 1.57)	0.28
≥6.21 (n=6056)	87		7	1.56 (0.72 to 3.38)	0.26		4	0.65 (0.24 to 1.77)	0.39		5	0.69 (0.28 to 1.70)	0.42
HDL cholesterol (mmol/l):													
<1.03 (4762)	60		5	1.56 (0.63 to 3.90)	0.34		7	1.55 (0.70 to 3.42)	0.28		6	1.33 (0.57 to 3.07)	0.51
1.03-1.54 (n=14 536)	121		13	2.19 (1.23 to 3.89)	0.008		5	0.61 (0.25 to 1.49)	0.28		7	0.81 (0.38 to 1.74)	0.60
≥1.55 (n=8221)	70		4	1.27 (0.46 to 3.48)	0.64		5	1.12 (0.45 to 2.79)	0.81		3	0.61 (0.19 to 1.95)	0.41

HDL=high density lipoprotein.

*Hazard ratio=1.

†History of migraine but no active migraine in year before completion of baseline questionnaire.

Table 5 summarises the association between migraine and myocardial infarction stratified by individual components of the Framingham risk score. The association between active migraine with aura and myocardial infarction was increased in the age groups 45-54 and 55-64. In contrast with ischaemic strokes, however, there was no further increased risk when we evaluated the age group 45-49 (data not shown). With regard to systolic blood pressure, we found an increased risk of myocardial infarction for women with active migraine with aura who had values <120 mm Hg and ≥140 mm Hg. While the risk did not further increase in categories above 140 mm Hg (data not shown), the associated risk of myocardial infarction was strongest for women with active migraine with aura who had a systolic blood pressure <110 mm Hg (10.00, 2.96 to 33.77; P<0.001). In contrast with ischaemic stroke, the association between active migraine with aura and migraine with aura increased with increasing total cholesterol concentrations. The pattern of association between active migraine with aura and myocardial infarction according to category of smoking was similar to the findings for ischaemic stroke.

**Table 5 tbl5:** Age adjusted association (hazard ratio) between migraine and myocardial infarction, stratified by components of Framingham risk score in 27 519 women in women’s health study

	No history of migraine (n=22 445)*		Active migraine with aura (n=1418)				Active migraine without aura (n=2159)				Previous migraine† (n=1497)		
			No of women	HR (95% CI)	P value		No of women	HR (95% CI)	P value		No of women	HR (95% CI)	P value
Age (years):													
45-54 (n=16 444)	65		10	2.10 (1.08 to 4.09)	0.03		7	0.92 (0.42 to 2.00)	0.83		7	1.75 (0.81 to 3.83)	0.16
55-64 (n=8160)	109		11	2.09 (1.12 to 3.90)	0.02		9	1.16 (0.58 to 2.28)	0.68		5	0.62 (0.25 to 1.52)	0.30
65 (n=2915)	66		3	1.39 (0.44 to 4.42)	0.58		1	0.46 (0.06 to 3.28)	0.43		8	1.65 (0.79 to 3.45)	0.18
Systolic blood pressure (mm Hg):													
<120 (n=12 389)	38		7	3.53 (1.57 to 7.95)	0.002		4	1.37 (0.48 to 3.84)	0.56		1	0.44 (0.06 to 3.17)	0.41
120-139 (n=11 678)	135		10	1.33 (0.70 to 2.53)	0.39		11	1.01 (0.54 to 1.87)	0.99		12	1.21 (0.67 to 2.18)	0.54
≥140 (n=3452)	67		7	2.45 (1.12 to 5.36)	0.03		2	0.45 (0.11 to 1.83)	0.26		7	1.34 (0.61 to 2.91)	0.46
Smoking:													
Never (n=14 219)	78		11	2.82 (1.49 to 5.32)	0.001		7	1.16 (0.53 to 2.53)	0.71		9	1.64 (0.82 to 3.27)	0.16
Past (10 100)	94		6	1.26 (0.55 to 2.88)	0.59		7	1.16 (0.53 to 2.51)	0.71		5	0.78 (0.32 to 1.91)	0.58
Current (n=3200)	68		7	2.08 (0.95 to 4.55)	0.07		3	0.65 (0.20 to 2.06)	0.46		6	1.17 (0.51 to 2.69)	0.72
Total cholesterol (mmol/l):													
<5.17 (n=11 171)	61		4	1.24 (0.45 to 3.41)	0.68		3	0.69 (0.21 to 2.20)	0.52		7	1.77 (0.81 to 3.87)	0.15
5.17-6.20 (n=10 292)	90		7	1.54 (0.71 to 3.34)	0.27		9	1.33 (0.66 to 2.65)	0.42		7	1.19 (0.55 to 2.56)	0.66
≥6.21 (n=6056)	89		13	2.71 (1.51 to 4.86)	<0.001		5	0.73 (0.30 to 1.80 )	0.49		6	0.82 (0.36 to 1.89)	0.65
HDL cholesterol (mmol/l):													
<1.03 (4762)	82		8	1.71 (0.83 to 3.55)	0.15		8	1.14 (0.55 to 2.38)	0.72		8	1.27 (0.62 to 2.63)	0.52
1.03-1.54 (n=14 536)	123		12	1.94 (1.07 to 3.52)	0.03		8	0.93 (0.45 to 1.91)	0.85		7	0.81 (0.38 to 1.72)	0.58
≥1.55 (n=8221)	35		4	2.44 (0.86 to 6.88)	0.09		1	0.42 (0.06 to 3.11)	0.40		5	2.07 (0.81 to 5.28)	0.13

HDL=high density lipoprotein.

*Hazard ratio=1.

†History of migraine but no active migraine in year before completion of baseline questionnaire.

## Discussion

### Summary of findings

In this large prospective cohort of women, the association between active migraine with aura and specific ischaemic vascular events differed with vascular risk status according to Framingham risk score groups. The association between active migraine with aura and ischaemic stroke was apparent only among women in the lowest Framingham risk score group, while the association with myocardial infarction was apparent only among women in the highest group. With regard to the individual components of the Framingham risk score, this diametric pattern of association was driven by particularly increased risk of ischaemic stroke among women with active migraine with aura who were young (aged 45-49) and who had low total cholesterol concentrations. In contrast, women with active migraine with aura who had high total cholesterol concentrations had an increased risk of myocardial infarction. The association between migraine with aura and coronary revascularisation procedures was similar to the myocardial infarction finding, while our data suggest a U shaped association between migraine with aura and angina according to the Framingham risk score.

We have previously shown that the association between migraine with aura and cardiovascular disease in the women’s health study remains significant even after adjustment for major cardiovascular risk factors.^11^ The data presented here suggest that among women with migraine with aura, information on the underlying vascular risk status can help to identify women who are at particular increased risk for specific cardiovascular disease events.

Our data add to the growing evidence that migraine is associated with increased risk of vascular events^6^ ^8^ ^11^ ^12^ and that the risk of specific cardiovascular disease outcomes might depend on the presence or absence of vascular risk factors.^5^ ^8^ Our results are of interest for general practitioners as a history of migraine in combination with information on vascular risk status, as measured by the Framingham risk score, could indicate future cardiovascular disease events. This is analogous to recent work indicating that a history of pre-eclampsia is a risk factor for cardiovascular disease in later life.^23^ Our data imply that among patients with migraine, cardiovascular risk factors should be more carefully sought and controlled.

Results of the genetic epidemiology of migraine study suggest that migraine, particularly migraine with aura, is associated with increased likelihood of an unfavourable cardiovascular risk profile and a high Framingham risk score.^13^ Compared with participants without migraine and predicted risk (according to the Framingham risk score) for coronary heart disease of <1%, migraineurs with aura had age and sex adjusted odds ratios of 1.84 (0.8 to 4.5) for predicted risk of 10-20%, and 4.01 (1.1 to 15.0) for predicted risk of ≥21%. While in our study any history of migraine was also associated with increased predicted 10 year risk of coronary heart disease (according to the Framingham risk score), this association did not differ with regard to migraine aura status. We had too few participants with very high Framingham risk scores, however, to allow us to evaluate the association between migraine and 10 year risk prediction for coronary heart disease of ≥21%.

### Potential biological mechanisms

Recent studies on the pathophysiology of migraine suggest that migraine can also be viewed in part as a systemic disorder that is affecting the vasculature, and migraineurs might have reduced number and function of endothelial progenitor cells, a surrogate for marker for impaired vascular function.^24^ Even in the absence of vascular risk factors, people with migraine have decreased cerebral^25^ and peripheral vascular resistance^3^ and increased likelihood of retinal microvascular signs,^4^ hypercoagulability,^26^ and inflammation.^27^ Moreover, the altered vascular reactivity is already present among young patients with a recent onset of migraine (mean age 24.6).^3^

Findings from previous studies have suggested that the association between migraine with aura and ischaemic stroke is more apparent for individuals without vascular risk factors^5^ ^7^ ^8^ with the exception of smoking and use of oral contraceptives in women.^8^ ^14^ In particular, people with migraine and no history of hypertension or diabetes had increased risk of ischaemic stroke.^5^ ^8^ People with migraine also have a higher likelihood of retinal microvascular signs.^4^ Furthermore, the proportion of ischaemic strokes among patients with migraine is less likely to be of atherosclerotic origin,^7^ ^8^ and, specifically, ischaemic strokes in the posterior circulation of migraineurs are probably caused by microembolism and hypoperfusion.^28^ The increased risk of ischaemic stroke for migraineurs with aura in the lowest Framingham risk score group seen in our study supports these findings. Because the score is composed of traditional risk factors for cardiovascular disease, it can be interpreted as a proxy for vascular health. Thus, it is plausible that the biological mechanisms by which migraine with aura results in ischaemic vascular events in the brain require a vasculature not altered by atherosclerosis but might involve microvascular changes.

Several studies have found an association between migraine with aura and angina but not myocardial infarction.^9^ ^10^ In addition, patients with active migraine with chest symptoms or suspected coronary ischaemia had lower angiographic coronary severity scores than patients with similar symptoms but no migraine.^29^ In our data, the association between migraine with aura and angina was increased for patients in the low and high risk score groups. Furthermore, our data indicate that the association between migraine with aura and myocardial infarction is apparent only among patients with a high Framingham risk score. Thus, the effect of migraine with aura on the coronary arteries might involve two mechanisms, one involving a vasculature not altered by atherosclerosis leading to angina and one involving a vasculature impaired by atherosclerosis leading to angina and myocardial infarction. Moreover, as women who reported a history of migraine but not active migraine at baseline (that is, the migraine had stopped or the frequency of migraine attacks was less than one attack a year) had a stronger association with high Framingham risk score and are also more likely to have increased levels of biomarkers of cardiovascular disease,^30^ we suggest that an impaired vasculature is involved in the declining one year prevalence of migraine in older age groups.^1^ As our data do not allow us to directly test this hypothesis, however, future research is warranted to further evaluate potential biological mechanisms of the association between migraine with aura and ischaemic vascular events. Of particular interest is whether continuing attacks of migraine with aura lead to changes in the peripheral vasculature, which increase the susceptibility to atherosclerotic changes.

### Strengths and weaknesses of study

Our study has several strengths, including the prospective design, large number of participants and outcome events, long follow-up and participation rate, standardised evaluation of migraine and cardiovascular disease risk factors, confirmed cardiovascular disease events by an end points committee of physicians, and the homogeneous nature of the cohort, which could reduce confounding.

Several limitations should be also considered when interpreting our results. Firstly, migraine and migraine aura status were self reported leading to potential misclassification. Because of the prospective design, however, any misclassification would probably result in underestimation of relative risks and would be unlikely to explain the observed association pattern. Furthermore, we have previously reported that our migraine classification had good agreement with 1988 IHS criteria^11^ and our prevalence of migraine and migraine aura is close to that reported in other population based studies.^1^ ^31^ ^32^ Secondly, we had no detailed information on use of specific drugs for migraine that might be associated with ischaemic events. Such drugs, however, tend to be used by all migraineurs, not just those with migraine aura. Furthermore, the different pattern of association between migraine with aura and ischaemic stroke and myocardial infarction according to vascular risk status can hardly be explained by drug use. Thirdly, despite the overall high number of outcome events, this number was small when we stratified by migraine and Framingham risk score categories. Fourthly, our stratified analyses are adjusted only for age and not other major cardiovascular disease risk factors. We have previously shown, however, that the association between migraine with aura and cardiovascular disease in this cohort is independent of major risk factors for cardiovascular disease.^11^ In addition, inclusion of major risk factors for cardiovascular disease as well as randomised treatment assignments in the stratified models showed similar associations (data not shown). Residual confounding remains a potential alternative explanation as our data are observational. Finally, participants in the women’s health study were all health professionals aged ≥45 and mostly white, thus generalisability might be limited. We have no reason to believe that that the biological mechanisms by which migraine with aura might be associated with vascular events would be different in other populations of women.

### Conclusions

Results from this large prospective study of women indicate that information on vascular risk status might help to identify women with migraine with aura who are at a particularly increased risk for specific future cardiovascular disease events with a diametric pattern of association for ischaemic stroke and myocardial infarction. Physicians should consider evaluating vascular risk factors in patients with migraine with aura.

**What is already known on this topic**

Migraine with aura is associated with increased risk of ischaemic stroke and other ischaemic vascular events
Migraine with aura is also associated with an unfavourable vascular risk profile as measured by the Framingham risk score for coronary heart disease

**What this study adds**

Migraine with aura is associated with increased risk of ischaemic stroke only among women with low vascular risk scores, while migraine with aura is associated with increased risk of myocardial infarction only among women with high vascular risk scores
Information on history of migraine and vascular risk status might help to identify women at increased risk for specific future cardiovascular disease events

## Notes

We thank the participants in the women’s health study for their outstanding commitment and cooperation and all the staff for their expert and unfailing assistance.

Provenance and peer review: Not commissioned; externally peer reviewed.

## Notes

**Cite this as:** *BMJ* 2008;337:a636
